# Study on the Protective Efficacy of the Japanese Encephalitis Live Attenuated Vaccine SA14-14-2 Against Newly Isolated Genotype I Japanese Encephalitis Viruses

**DOI:** 10.3390/v18050582

**Published:** 2026-05-21

**Authors:** Shuai Shang, Qikai Yin, Tingyi Che, Xinzhu Wang, Qi Su, Shihong Fu, Hongshan Xu, Yongxin Yu, Qunying Mao, Huanyu Wang, Xinyu Liu

**Affiliations:** 1 National Key Laboratory of Drug Regulatory Science, National Institutes for Food and Drug Control, Beijing 102629, China; shangshuai2026@163.com (S.S.); euemsh@foxmail.com (T.C.); wupcp73@163.com (X.W.); rivaille_sq@foxmail.com (Q.S.); xuhongshan@nifdc.org.cn (H.X.);; 2 Changchun Institute of Biological Products Co., Ltd., Changchun 130015, China; 3 State Key Laboratory of Novel Vaccines for Emerging Infectious Diseases, Beijing 100024, China; 4 National Key Laboratory of Intelligent Tracking and Forecasting for Infectious Diseases, National Institute for Viral Disease Control and Prevention, Chinese Center for Disease Control and Prevention, Beijing 102206, China

**Keywords:** Japanese encephalitis virus (JEV), genotype I (GI), genotype III (GIII), SA14-14-2 vaccine, live attenuated vaccine (LAV), cross-protection, genotype shift, neutralizing antibodies

## Abstract

Japanese encephalitis virus (JEV) comprises a single serotype but can be classified into five genotypes (genotypes I–V, GI–GV) based on nucleic acid sequences. Historically, genotype III (GIII) was the predominant strain. However, since the 21st century, genotype I (GI) rapidly replaced GIII as the major genotype in China, Southeast Asia, and other regions. The live attenuated vaccine (LAV) SA14-14-2, licensed in China in 1988, was successfully exported to 13 countries, with cumulative vaccinations exceeding one billion doses. The vaccine seed virus SA14-14-2 belonged to genotype III. Whether this GIII-based vaccine provided sufficient protection against the currently circulating GI strains warranted systematic investigation. In this study, recent JEV isolates collected from China were subjected to genotypic analysis, followed by comprehensive evaluations including protective efficacy against challenge and serum neutralizing antibody levels. The results indicated that, despite antigenic differences between GIII and GI strains, no significant differences in protective efficacy post-challenge were observed. The SA14-14-2 LAV remained effective in preventing GI strain infection.

## 1. Introduction

Japanese encephalitis virus (JEV) is a mosquito-borne *flavivirus* transmitted primarily by *Culex tritaeniorhynchus mosquitoes* [1]. It remains a leading cause of viral encephalitis in Asia, with mainland China accounting for approximately 50% of globally reported cases [2]. The virus belongs to the genus Orthoflavivirus within the family Flaviviridae. It is an enveloped, positive-sense, single-stranded RNA virus with a genome of approximately 11 kilobases, encoding three structural proteins (capsid protein C, precursor membrane protein prM, and envelope protein E) and seven non-structural proteins (NS1, NS2A, NS2B, NS3, NS4A, NS4B, and NS5). The envelope (E) protein plays a critical role in receptor binding and mediating viral entry into host cells, and serves as the primary target of neutralizing antibodies [3,4]. Although JEV comprises only a single serotype, phylogenetic analysis based on nucleotide sequences classifies it into five genotypes (genotypes I–V, GI–GV) [5]. Historically, genotype III (GIII) is the long-standing predominant genotype across Asia. However, since the early 21st century, genotype I (GI) gradually replaces GIII as the major epidemic strain circulating in China and Southeast Asia. This shift may be attributed to enhanced adaptation of GI strains to avian hosts, characterized by higher viremia levels and a reduced capacity to induce interferon responses [6,7].

This genotypic shift implies potential antigenic differences between newly isolated GI strains and the GIII-derived SA14-14-2 live attenuated vaccine (LAV), which could affect vaccine efficacy. Since its approval in China in 1988, the SA14-14-2 LAV has been successfully exported to 13 countries, with cumulative administrations exceeding one billion doses. Numerous studies confirm its excellent safety and immunogenicity [8,9,10,11,12]. Our previous research demonstrates significant protection against GI strains isolated around the year 2000 [13]. However, some recent studies suggested that GIII-based vaccines might not provide effective protection against newly isolated GI strains [14].

Accordingly, this study aimed to assess the protective efficacy of the SA14-14-2 live attenuated vaccine against six recently isolated JEV strains from China. These findings were intended to provide a scientific basis for the continued rational use of this vaccine.

## 2. Materials and Methods

### 2.1. Ethical Statement

All procedures involving animals were approved by the Ethics Committee on Animal Research of the National Institutes for Food and Drug Control (NIFDC), Beijing, China under protocol number No. NIFDC (F) 2025(B)093 and FB2025117. The animal immunization procedures adhered to international guidelines and Chinese law.

### 2.2. Virus Strains

Six new isolated JEV strains (LN1821, LN1864, HNDZ1751-2, ZJ18-66, ZJ2021SY495, ZJ2022WY838), isolated in China between 2018 and 2022, were provided by Professor Huanyu Wang from the National Institute for Viral Disease Control and Prevention, Chinese Center for Disease Control and Prevention, China CDC. The GIII strain P3 (isolated in Beijing in 1949) was used as a control. All viruses were stored in National Institutes for Food and Drug Control (Table 1).

### 2.3. Cells, Mice and Vaccine

BHK-21 cells were cultured in DMEM containing 10% fetal bovine serum (FBS) and 1% penicillin–streptomycin (PS). BHK-21 cells were incubated at 37 °C in a humidified atmosphere with 5% CO_2_, and all media and reagents were obtained from Gibco (Carlsbad, CA, USA).

Three-week-old specific-pathogen-free Kunming mice (equal numbers of males and females), weighing between 12 and 14 g, were obtained from the Institute of Laboratory Animal Resources, National Institutes for Food and Drug Control (NIFDC), Beijing, China.

The commercial SA14-14-2 LAV used for immunization was obtained from Chengdu Institute of Biological Products Co., Ltd., Chengdu, China.

### 2.4. Viral RNA Extraction and Sequencing

#### 2.4.1. Lysis and Binding

For each of the six new isolated JEV strains (LN1821, LN1864, HNDZ1751-2, ZJ18-66, ZJ2021SY495, ZJ2022WY838) and the P3 strain, 140 μL of viral sample was added to a 1.5 mL microcentrifuge tube. Then, 560 μL of Buffer AVL containing carrier RNA was added to each tube. The mixture was pulse-vortexed for 15 s and incubated at room temperature for 10 min. After brief centrifugation, 560 μL of absolute ethanol was added to each tube, followed by pulse-vortexing for 15 s and another brief centrifugation.

#### 2.4.2. Loading and Washing

A 630 μL aliquot of the mixture was applied to a QIAamp Mini spin column (QIAGEN, Hilden, Germany). The column cap was closed and centrifuged at 6000× *g* for 1 min. The column was then placed into a new 2 mL collection tube, and the old tube with the flow-through was discarded. This step was repeated until all of the mixture had been loaded. Then, 500 μL of Buffer AW1 was added to the column, centrifuged at 6000× *g* for 1 min, and the column was transferred to a new 2 mL collection tube, discarding the old tube and flow-through. Then, 500 μL of Buffer AW2 was added, and the column was centrifuged at 12,000× *g* for 3 min. Finally, the column was placed in a new 2 mL collection tube and centrifuged at full speed for 1 min to completely remove any residual Buffer AW2.

#### 2.4.3. Elution and Sequencing

The column was transferred to a clean, RNase-free 1.5 mL microcentrifuge tube, discarding the old collection tube. Sixty microliters of Buffer AVE, equilibrated to room temperature, was added to the center of the column membrane. The cap was closed, and the column was incubated at room temperature for 1 min, followed by centrifugation at 6000× *g* for 1 min to collect the eluate. The extracted viral RNA was sent to a sequencing company (Sangon Biotech Co., Ltd., Shanghai, China) for whole-genome sequencing and analysis.

### 2.5. Phylogenetic and Genetic Distance Analysis

Genetic distance analysis and phylogenetic tree construction were performed using MEGA 12 software based on the complete genome sequences of each newly isolated strain, the SA14-14-2 LAV strain, the P3 strain, the K94P05 strain (gene bank accession number: AF045551), the FU strain (gene bank accession number: AF217620), the JKT6468 strain (gene bank accession number: AY184212), the XZ0934 strain (gene bank accession number: JF915894), and West Nile virus (gene bank accession number:DQ318020.1, as an outgroup).

#### 2.5.1. Genetic Distance Analysis

The genetic distance matrix following sequence alignment was calculated and constructed using MEGA 12 software (https://www.megasoftware.net, accessed on 25 November 2025). The substitution model was selected as follows: nucleotide-level substitutions were used for the type of nucleotide replacement, and evolutionary distances were corrected using the Maximum Composite Likelihood (MCL) model. Both transitions and transversions were included in the statistical scope. The site evolution pattern was set to uniform rates among sites, and the substitution pattern was assumed to be homogeneous across lineages. Gaps and missing data in the aligned sequences were handled using the pairwise deletion mode, meaning that missing sites were excluded only for each pair of taxa during distance calculation, thereby maximizing the retention of valid genetic information. For the analysis of site selection, coding positions (first, second, and third codon positions) as well as noncoding sites were comprehensively included. Distance calculation was performed using the pairs of taxa mode to compute pairwise genetic distances between all samples, ultimately generating a symmetric genetic distance matrix. The variance estimation method was set to “None” for this study.

#### 2.5.2. Phylogenetic Tree Construction

A maximum likelihood (ML) phylogenetic tree was constructed using the Tamura–Nei (TN93) nucleotide substitution model, with 1000 replicates of standard bootstrap analysis. The evolutionary rate among sites was set to uniform rates, and all codon positions (first, second, and third) as well as noncoding sites were included in the analysis. All gaps and missing data sites in the aligned sequences were retained. ML tree topology search was performed using the nearest-neighbor interchange (NNI) heuristic algorithm. The initial tree for ML optimization was automatically generated using the neighbor-joining (NJ) method, with no branch-swapping filter applied. The calculation was performed using eight-thread CPU parallel processing.

### 2.6. Plaque Reduction Neutralization Test (PRNT)

Three-week-old specific-pathogen-free (SPF) female Kunming mice (12–14 g) were immunized with a single intraperitoneal (i.p.) dose of SA14-14-2 LAV at 10^−3^ dilution (3000 PFU) per mouse. Pooled sera were collected 14 days after primary immunization for use in neutralization assays. Pooled serum samples from unvaccinated mice served as negative controls.

Each of the seven challenge viruses were incubated with serial two-fold dilutions (from 1:5 to 1:80) of either the LAV-immunized mouse sera or the control serum at a 1:1 ratio for 90 min at 37 °C. The virus-serum mixtures were then added to confluent monolayers of BHK-21 cells and incubated for 1 h. Subsequently, the inoculum was replaced with an overlay medium containing 1% methylcellulose, and the cells were cultured for an additional 5 days. Plaques were visualized by crystal violet staining and counted. The neutralizing antibody titer was defined as the reciprocal of the highest serum dilution that resulted in a ≥50% reduction in plaque count compared to the control serum from unvaccinated mice.

### 2.7. Mouse Immunization and Challenge

Three-week-old specific-pathogen-free (SPF) Kunming mice weighing 12–14 g (equal numbers of males and females) were administered a single dose of LAV via intraperitoneal injection at doses of 3000 PFU, 300 PFU, and 30 PFU per mouse (10 mice per group). Control mice were injected with PBS. Fourteen days post-immunization, the mice were challenged with a lethal dose (approximately 1000 LD50) of each Japanese encephalitis virus strain via combined intracranial injection of 0.3 mL of virus and 0.02 mL of PBS. Survival and morbidity rates were recorded over a 14-day observation period. All animal experiments were approved by the Animal Welfare and Ethics Committee.

## 3. Results

### 3.1. Phylogenetic Analysis Reveals Significant Genetic Distances Between Genotypes

**Figure 1:**
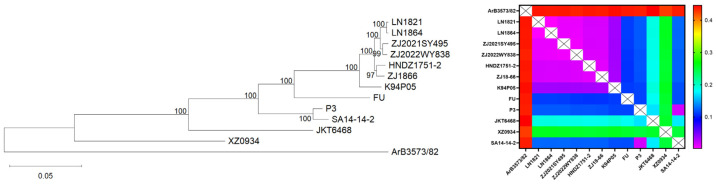
Phylogenetic tree based on complete viral genome sequences. (LN1821, LN1864, ZJ2021SY495, ZJ2022WY838, HNDZ1751-2, and ZJ18-66 are newly isolated JEV strains; SA14-14-2 is a GIII vaccine strain; K94P05, FU, P3, JKT6468, and XZ0934 are GI–GV strains, respectively, from NCBI, used to differentiate genotypes; ArB3573/82 is a West Nile virus strain used to confirm the identity of the 12 aforementioned strains as JEV). The integration of the phylogenetic tree with the genetic distance heatmap provided a comprehensive view of virus evolution. The heatmap not only confirmed the monophyly of each major clade defined by the tree but also highlighted the extent of divergence within Clade 1 versus Clade 2.

Phylogenetic analysis was performed based on complete genome sequences, including the West Nile virus strain (as an outgroup), six newly isolated GI strains, the P3 control strain, the SA14-14-2 vaccine strain, and representative strains of JEV genotypes I, II, IV, and V obtained from GenBank. Based on the calculated genetic distance matrix, a genetic distance heatmap and a phylogenetic tree were generated (Table 2 and Figure 1). The results showed that the six newly isolated JEV strains belonged to genotype I, and the genetic distance matrix confirmed a high degree of homogeneity within the GI cluster. The topology of the phylogenetic tree clearly indicated that the challenge viruses formed a genetically coherent group that was evolutionarily distinct from the vaccine strain.

### 3.2. Neutralizing Efficacy of Vaccine Immune Sera Against Newly Isolated Strains

The SA14-14-2 LAV induced moderate PRNT50 titers (ranging from 1:10 to 1:20) in immunized mice against the six newly isolated GI strains. In contrast, a higher titer (1:40) was observed against the GIII P3 strain (Table 3).

### 3.3. Protective Efficacy of SA14-14-2 LAV Against Newly Isolated Strains

A single dose of SA14-14-2 LAV provided excellent protection against all seven JEV strains (Table 4). At the highest vaccine dose (3000 PFU), complete protection (100% survival) was observed against each GI and GIII strain. Even at the lowest vaccine dose (30 PFU), protective efficacy remained ≥90%. Compared to the GIII P3 control strain, survival rates against GI strains did not show a consistent decline. To explore the relationship between genetic distance and immunogenicity, we compared the neutralizing titers, protective efficacy, and pairwise genetic distance from the SA14-14-2 vaccine strain for each challenge virus (Table 5).

Integrated analysis revealed a clear trend: all GI strains had substantially greater genetic distances from the vaccine strain (all ≥ 0.124) compared to the GIII P3 strain (0.018). This greater genetic divergence results to the antigenic differences and a 2-to 4-fold reduction in neutralizing antibody titers against GI strains. Most importantly, however, this reduction in in vitro neutralizing capacity did not result in a decrease in in vivo protective efficacy upon challenge.

## 4. Discussion

Since 1990s, genotype I (GI) Japanese encephalitis virus has rapidly replaced genotype III (GIII) as the predominant genotype in Asia [15]. The SA14-14-2 LAV, widely used in China, is derived from a GIII strain. Whether this vaccine remains still effective against currently circulating GI strains is a key scientific question that urgently needs to be addressed in vaccine use.

In this study, we systematically evaluated the protective efficacy of the SA14-14-2 LAV against six GI JEV strains isolated in China between 2017 and 2022. Immunized mice were challenged via intraperitoneal inoculation combined with intracranial injection of PBS. The results showed that at the highest immunization dose of 3000 PFU, the vaccine provided 100% protection against all GI strains. Even at the lowest dose of 30 PFU (approximately 1/100,000 of the human immunization dose), the protection rate remained ≥90%. These findings clearly demonstrated that the SA14-14-2 LAV retains excellent protective efficacy against recently isolated GI JEV strains, which is highly consistent with our previous findings [13].

In this study, the neutralizing titers of sera from SA14-14-2-immunized mice against GI strains were 2–4 times lower than those against the GIII P3 strain. This reduction was speculated to be associated with approximately 12% nucleotide and 3% amino acid sequence differences in the E protein between GI and GIII JEV strains [16]. Nevertheless, the PRNT_50_ titers of sera against all six GI strains were ≥1:10, still reaching the effective protective threshold generally accepted.

The world’s first inactivated Japanese encephalitis vaccine was developed in Japan in 1954 using the GIII Nakayama strain. It is reported in 1994 that GI was gradually replacing GIII as the dominant circulating strain in Japan [17]. However, previous studies confirmed that although the neutralizing antibody titers against GI strains in human sera following immunization with the Nakayama strain inactivated vaccine were lower than those against the GIII strain, the seroconversion rates still remained high demonstrating enough efficacy against GI strains [18,19].

A previous study also noted that since GI replaced GIII as the dominant strain in Japan, no significant increase in the morbidity of Japanese encephalitis has been observed, suggesting that the currently used GIII vaccines still provide enough protection against the circulating GI strains [15,17,20].

In China, GI has gradually replaced GIII as the dominant genotype since 2000. But after the Japanese encephalitis vaccine added into China’s National Expanded Immunization Program in 2008, the Japanese encephalitis cases has gradually decreased to approximately 200, the lowest level in history [21]. It demonstrates that the GIII-based vaccine can still provide good protective efficacy against the currently circulating GI strains. It confirmes that although the neutralizing antibody titers against GI induced by the GIII vaccine are reduced, they can still provide effective protection.

It was also noteworthy in this study that despite significant differences between GI strains and the vaccine strain in genetic distance and neutralizing titers, there was no marked decline of protective efficacy in mice. Even low-dose immunization (30 PFU) provided a high level of protection, suggesting that the protective mechanism of this vaccine was not dependent solely on neutralizing antibodies. Our previous research confirmed that this vaccine induced a strong and persistent Th1-type cellular immune response, with high-level IFN-γ and IL-2 [22]. Additionally, it induced specific T-cell responses against nonstructural proteins such as JEV NS3, and the NS1 protein could induce agglutinating antibodies as well as antibody-dependent cellular cytotoxicity (ADCC) responses, thereby providing robust protection even at low immunization doses [23,24,25,26].

In summary, the findings of this study clearly demonstrate that the SA14-14-2 live attenuated Japanese encephalitis vaccine exhibits excellent protective efficacy against recently isolated GI strains in China. These results strongly support the continued use of the GIII-based SA14-14-2 live attenuated vaccine for the prevention and control of Japanese encephalitis in areas where GI strains are currently circulating.

## Figures and Tables

**Table 1:** JEV used in this study.

No.	Strain	Accession Number	Isolation Source	Isolation Time	Isolation Cell Line	Location of Collection	lgPfu/LD_50_	Genotype
1	LN1821	OM572547.1	Mosquito	2018	C6/36	Liaoning	2.82	I
2	LN1864	/	Mosquito	2018	C6/36	Liaoning	2.58	I
3	ZJ2021SY495	/	Mosquito	2017	C6/36	Zhanzhou, Hainan	3.49	I
4	ZJ2022WY838	/	Mosquito	2018	C6/36	Zhejiang	3.98	I
5	HNDZ1751-2	KX779520.1	Mosquito	2021	C6/36	Songyang, Lishui, Zhejiang	1.99	I
6	ZJ18-66	OM572549.1	Mosquito	2020	C6/36	Wuyi, Jinhua, Zhejiang	3.69	I
7	P3	/	Human brain	1949	Mouse brain	Beijing	0.88	III

**Table 2:** Genetic distance matrix based on complete genome sequences of newly isolated JEV strains.

Genotype	/	I	I	I	I	I	I	I	II	III	IV	V	III
JEV Strains	ArB3573/82	LN1821	LN1864	ZJ2021SY495	ZJ2022WY838	HNDZ1751-2	ZJ18-66	K94P05	FU	P3	JKT6468	XZ0934	SA14-14-2
Serial Number	A	B	C	D	E	F	G	H	I	J	K	L	M
**A**		0.439	0.438	0.438	0.435	0.438	0.437	0.442	0.437	0.435	0.446	0.425	0.436
**B**	0.439 *		0.003	0.010	0.010	0.017	0.016	0.035	0.112	0.122	0.190	0.258	0.127
**C**	0.438	0.003		0.010	0.009	0.016	0.016	0.035	0.112	0.122	0.190	0.258	0.127
**D**	0.438	0.010	0.010		0.007	0.016	0.016	0.035	0.111	0.122	0.188	0.257	0.127
**E**	0.435	0.010	0.009	0.007		0.015	0.015	0.034	0.109	0.120	0.188	0.255	0.124
**F**	0.438	0.017	0.016	0.016	0.015		0.012	0.033	0.108	0.120	0.186	0.252	0.125
**G**	0.437	0.016	0.016	0.016	0.015	0.012		0.032	0.108	0.119	0.186	0.254	0.124
**H**	0.442	0.035	0.035	0.035	0.034	0.033	0.032		0.111	0.115	0.192	0.259	0.118
**I**	0.437	0.112	0.112	0.111	0.109	0.108	0.108	0.111		0.114	0.184	0.254	0.118
**J**	0.435	0.122	0.122	0.122	0.120	0.120	0.119	0.115	0.114		0.171	0.249	0.018
**K**	0.446	0.190	0.190	0.188	0.188	0.186	0.186	0.192	0.184	0.171		0.255	0.174
**L**	0.425	0.258	0.258	0.257	0.255	0.252	0.254	0.259	0.254	0.249	0.255		0.249
**M**	0.436	0.127	0.127	0.127	0.124	0.125	0.124	0.118	0.118	0.018	0.174	0.249	

* A pairwise genetic distance matrix based on complete genome sequences is presented in Table 2. The values represent the p-distance, indicating the proportion of nucleotide differences between strains.

**Table 3:** Neutralizing antibody titers against different Japanese encephalitis virus strains.

No.	Strain	Genotype	Neutralizing Antibody Titer (PRNT_50_)
1	LN1821	I	1:10
2	LN1864	I	1:10
3	HNDZ1751-2	I	1:20
4	ZJ18-66	I	1:10
5	ZJ2021SY495	I	1:10
6	ZJ2022WY838	I	1:10
7	P3	III	1:40

**Table 4:** Protective efficacy against challenge with different Japanese encephalitis virus strains.

No.	Strain	Genotype	Survival Rate				Challenge Dose (LD_50_)
			3000 PFU	300 PFU	30 PFU	Control	
1	LN1821	I	10/10 *	10/10	10/10	2/10	3.20 lg **
2	LN1864	I	10/10	10/10	10/10	0/10	3.23 lg
3	HNDZ1751-2	I	10/10	10/10	10/10	2/10	2.85 lg
4	ZJ18-66	I	10/10	10/10	10/10	0/10	3.15 lg
5	ZJ2021SY495	I	10/10	10/10	9/10	2/10	2.76 lg
6	ZJ2022WY838	I	10/10	9/10	10/10	2/10	3.16 lg
7	P3	III	10/10	10/10	10/10	2/10	3.12 lg

*: Number of survivors/total number; **: Challenge dose is 3.20 lg LD_50_.

**Table 5:** Neutralizing titer, protective efficacy, and pairwise genetic distance from the SA14-14-2 vaccine strain for each virus.

Virus Strain	Genotype	Genetic Distance from SA14-14-2	LAV PRNT50 Titer	Survival Rate (10^−5^ LAV)
HNDZ1751-2	I	0.125	1:20	10/10
ZJ18-66	I	0.124	1:10	10/10
LN1821	I	0.127	1:10	10/10
LN1864	I	0.127	1:10	10/10
ZJ2021SY495	I	0.127	1:10	9/10
ZJ2022WY838	I	0.124	1:10	10/10
P3	III	0.018	1:40	10/10

## Data Availability

The original contributions presented in this study are included in the article. Further inquiries can be directed to the corresponding authors.
